# A pharmacodynamic investigation to assess the synergism of orbifloxacin and propyl gallate against *Escherichia coli*

**DOI:** 10.3389/fphar.2022.989395

**Published:** 2022-09-15

**Authors:** Muhammad Aleem Abbas, Eon-Bee Lee, Naila Boby, Biruk Tesfaye Biruhanu, Seung-Chun Park

**Affiliations:** ^1^ Laboratory of Veterinary Pharmacokinetics and Pharmacodynamics, Cardiovascular Institute, College of Veterinary Medicine, Kyungpook National University, Daegu, Gyeongsangbuk-do, South Korea; ^2^ Cardiovascular Research Institute, Kyungpook National University School of Medicine, Daegu, Gyeongsangbuk-do, South Korea

**Keywords:** synergistic antimicrobial combinations, antibiotics resistance, Orbifloxacin, propyl gallate, *Escherichia coli*, Synergism, phenolic compound, pharmacodynamic model

## Abstract

*Escherichia coli* (*E. coli*) infections are becoming increasingly difficult to treat, as antibiotic-resistant variants proliferate. Studies on novel methods to combat the spread of resistance and improve the performance of current antibiotics are vital. We aimed to boost the efficacy of the antibiotic orbifloxacin (ORB) against *E. coli* by combining it with a phenolic component, propyl gallate (PG). The minimum inhibitory concentration (MIC) and minimum bactericidal concentration (MBC) of ORB against the *E. coli* KVCC 1423 resistant strain were 128 μg/ml and 256 μg/ml, respectively. However, the MIC of ORB for the remaining *E. coli* strains was 0.5 μg/ml–2 μg/ml. For the combination of PG and ORB, the lowest fractional inhibitory concentration (FIC) index was less than 0.5, and the combination decreased the MIC of both drugs by 74%. The time-kill assay revealed the killing properties of both the drugs and the pharmacodynamic model (PD model) confirmed the strong killing properties of the combination as compared to the individual activities of the drugs. The ratio between MIC and mutant prevention concentration of ORB against *E. coli* 1400306 and 1,423 were 1:32 and 1:8, respectively. The combination of ORB and PG showed strong biofilm eradication and inhibited the motility of bacteria. The cell viability of the combination was > 80%. Therefore, we believe that ORB and PG in combination could be a possible antibacterial candidate that could minimize resistance and improve antibiotic potential.

## 1 Introduction

Multidrug resistance in *E. coli* has become a worrisome problem that is increasing in human as well as veterinary medicine worldwide. Antibiotic resistance in *E. coli* is rapidly increasing, particularly against broad-spectrum antibiotics like fluoroquinolones (Collignon, 2009; Poirel et al., 2018). *E. coli*, is a gram-negative, facultatively anaerobic, rod-shaped, coliform bacterium of the genus *Escherichia* which is generally found in the lower gut of organisms (Singleton, 2004; Tenaillon et al., 2010). *E. coli* and other non-pathogenic facultative anaerobes compose approximately 0.1% of gut microbiota (Eckburg et al., 2005). Pathogenic *E. coli* can cause diarrhea when ingested *via* contaminated food or water and is associated with food poisoning; additionally, it can cause pneumonia and urinary tract infections, and 75%–95% of urinary tract infections are caused by *E. coli* (Mehnert-Kay, 2005; Yayan et al., 2015; Moon et al., 2022). Some strains of *E. coli* produce toxins like Shiga, which damage the intestinal mucosa (Schüller, 2011). It is challenging to control microorganisms because they constantly develop complex resistance against antibacterial agents (Khalil et al., 2021). Moreover, the medical treatment of diseases caused by pathogenic *E. coli* is challenged by the speedy development and spread of antibiotic-resistant strains (Qiu et al., 2019).

Broad-spectrum antibiotics are the first line of defense against bacterial infections. The loss of effectiveness of antimicrobials resulting from antimicrobial usage is a global public health dilemma. It is a challenging issue for many interconnected biological and societal reasons (Eltholth et al., 2022). Pathogenic illnesses caused by antibiotic-resistant bacterial strains cause active and passive damage in the livestock industry. Active losses include increased livestock deaths and lower livestock productivity, whereas passive losses include food insecurity, decreased market potential, loss of trade, and control costs (Wiethoelter et al., 2015). Approximately 75% of infectious illnesses are passed directly from animals to humans, which reflects a serious public health concern (Yasmeen et al., 2021; Song et al., 2022). According to researchers by 2050, the meat sector would need to increase its production by 50%–100% to meet the increasing population demand. The global meat output reached 263 million tons in 2018 and is projected to increase to 445 million tons by 2050 (Srutee et al., 2021). The serious issue to achieving these targets are bacterial diseases and emerging resistance in bacteria which directly affect production. As the result, antibiotic abuse is commonly practiced in the livestock industry to meet targets and overcome resistance to infectious pathogens, and drug residue from animal products is another challenge for humans.

Combining antibacterial drugs and natural products or syntactic agents may be a unique strategy for improving the efficacy of the already available antibiotics in inhibiting multidrug-resistant types of bacteria like *E. coli* (Ayaz et al., 2019; Hossain et al., 2020). Fluoroquinolones have been used in veterinary medicine since the 1980s. Orbifloxacin, a derivative of sparfloxacin, was created as a veterinary chemotherapeutic medication specifically for the treatment of gastrointestinal and respiratory diseases in cattle and domestic pets in 1987. Orbifloxacin (ORB), a 3rd generation fluoroquinolone, has enhanced antibacterial action against both gram-negative and gram-positive bacteria, high oral bioavailability, and prolonged terminal elimination half-life (better systemic distribution). It is considerably less hazardous to the central nervous system and has fewer interactions with the cytochrome P450 (CYP 450) system (Ball, 2000; Cazedey and Salgado, 2013). However, *E. coli* resistance against quinolones (orbifloxacin) is increasing (Stapleton et al., 2020). The resistance rate against quinolones in swine isolates from Europe, Canada, and Japan was 0%–39%, and it was very high in isolates from China and Brazil (81% and 54.4%, respectively) (Stapleton et al., 2020; Amsler et al., 2021). The reported minimum inhibitory concentration range of orbifloxacin was 0.3 µg–4 µg or 0.13 µg–128 µg (Gebru et al., 2011; Shimizu et al., 2017). Due to the emerging resistance against orbifloxacin, it is necessary to find a combination compound that increases the effect of the drug while reducing the quantity used.

In recent years naturally occurring polyphenol compound gallic acid (GA) and its derivatives are reported for their various biological activities comprising anticancer, anti-inflammatory, and antibacterial properties as well as modulating immunity. GA is abundantly present in several plants in different forms, and its derivative phenolic compound, propyl gallate (PG) (propyl 3, 4, 5-trihydroxybenzoate), is known for its best antioxidant properties (Yang et al., 2020). PG is not harmful to veal calves, growing cattle, milk cows, sheep, goats, piglets, equine, salmonids, ornamental fish, and dogs, and the use of PG in the animal diet has no environmental hazard (Additives et al., 2020). Hence, we aimed to investigate the antibacterial properties of PG and ORB against *E. coli*. Based on past studies and the features of PG outlined above, we hypothesized that PG may have the ability to boost the efficacy of ORB which will help to treat the resistant strains. However, to the best of our knowledge, no study has investigated the synergism between PG and ORB. In this study, the antibacterial properties of both compounds were tested individually and in combination against *E. coli*. Furthermore, the effects of this combination on pathogenic variables like motility and biofilm formation were evaluated. Finally, the effects of PG and ORB on cell survival, both alone and in combination, were investigated.

## 2 Materials and methods

### 2.1 Chemicals

Orbifloxacin was purchased from Sigma-Aldrich Co. (St. Louis, United States). PG was purchased from the Tokyo chemicals industry (TCI Co. Ltd., Tokyo Japan). The bacterial growth media, Mueller Hinton II and Luria Bertani (LB), were purchased from Difco, United States . All analytical grade chemicals and reagents were used in this study.

### 2.2 *Escherichia coli* strains, culture conditions, and media

*E. coli* (*E. coli*-KVCC-BA 0001423, *E. coli*-KVCC-BK 0000543, *E. coli*-KVCC-BA 1400306) were obtained from the National Veterinary Research and Quarantine Service (Gimcheon, Korea) (Kim et al., 2021). *Escherichia coli* ATCC 35218 was used as a quality control strain. All the *E. coli* were cultured in LB broth/agar (Difco, United States) at 37°C. The bacteria were grown overnight in LB broth at 37°C in a shaking incubator before the experiments. Mueller Hinton II (Cation adjusted) (Difco, United States) was used in all the experiments with antibacterial agents.

### 2.3 Detection of enterotoxigenic *Escherichia coli* by multiplex PCR

For the detection of enterotoxigenic *E. coli,* we have followed manufacturer instructions using a Multiplex PCR kit (RapiGEN, Cat. No.RDM-1101). Briefly, one ml of an overnight culture from the three E. c*ol*i strains (E. coli-KVCC-BA 0001423, E. coli-KVCC-BK 0000543, and E. coli-KVCC-BA 1400306) was transferred to each Eppendorf tube subsequently followed by centrifugation (at 13,000 rpm for 3 min), washing and DNA extraction. The extracted DNA was transferred to PCR tubes with 10 µl of premix given in the kit and processed according to the given PCR conditions. Finally, PCR was performed at 95°C (3 min) for pre-denaturation, followed by 40 cycles of 94°C (20 s) for denaturation, 63°C (25 s) for annealing, 72°C (45 s) for extension, and 72°C (5 min) for final extension. The final PCR product was loaded into a gel electrophoresis system at 100 v for 30 min and visualized by using a luminescent image analyzer (Image Quant-LAS500) by EAGLE-EYE.

### 2.4 Minimum inhibitory concentration and minimum bactericidal concentration determination

The MIC of ORB and PG were determined using the broth microdilution method following Clinical and Laboratory Standards Institute guidelines (CLSI, 2018). Briefly, two-fold serial dilutions of ORB and PG were prepared in Mueller–Hinton II Broth (MHB) (MHB II-Difco-BD, United States) using 96-well plates. The initial concentration of ORB against all the strains was 1,024 μg/ml. The overnight culture was diluted and adjusted to a concentration of 10^5^ colony-forming units (CFU/ml) of the *E. coli* strains and was dispensed to the designated wells of the 96-well plates. The plates were incubated for 18 h–20 h at 37°C, and the results were read with a microplate reader (Biotech EPOCH2, United States) at 600 nm. To determine the MBC, 20 μl of the microtiter plate suspension from the MIC was plated out on LB agar. The plates were incubated at 37°C for 24 h to identify potentially slow-growing bacteria. The test was performed in triplicates.

### 2.5 Time-kill assay

The time-kill assay was performed as previously reported, with minor modifications (Birhanu et al., 2021). To acquire bacteria in the logarithmic growth phase, the *E. coli* was inoculated was cultured into a 5 ml LB broth, and incubated at 37°C. The final bacterial concentrations of 10^5^ and 10^9^ CFU/ml of bacteria were prepared and treated with MIC, MBC, and 1/4 MIC of the drugs and incubated at 37°C. Sampled at 0 h, 1 h, 2 h, 4 h, 6 h, 12 h, and 24 h, and cultured on LB plates for 48 h after being serially diluted. After bacterial counting, the findings were recorded.

### 2.6 Fractional inhibitory concentration (FIC) of orbifloxacin and propyl gallate

The effects of the combination of ORB and PG on resistance in *E. coli* 1,423 were investigated using a previously published modified checkerboard microdilution technique (Hossain et al., 2020). To compare ORB and PG, one drug was applied horizontally and the other was applied vertically in serial dilutions in the wells of a 96-well plate to generate combinations of these two antibacterial agents at different ratios. Each test plate also contained individual dilutions of these antibacterial agents as well as drug-free control wells (medium only). *E. coli* cells in their first log phase were diluted, and 100 µL of these diluted suspensions were placed into each well of the 96-well plates, resulting in a final density of 10^5^ CFU/ml. These bacterial cells were cultured at 37°C for 18 h in various antibacterial solutions in the 96-well plates. The FIC and fractional inhibitory concentration index (FICI) were computed using the below formula below.

$$FIC\;of\;drug\;A=\frac{MIC\;of\;drug\;A\;in\;presence\;of\;drug\;B}{MIC\;of\;Drug\;A\;\left(Alone\right)}$$ (1)

$$FIC\;of\;drug\;B=\frac{MIC\;of\;drug\;B\;in\;presence\;of\;drug\;A}{MIC\;of\;Drug\;B\;\left(Alone\right)}$$ (2)

FIC Index=FIC of drug A+FIC of drug B (3)

### 2.7 Mutant prevention concentration assay

The MPC for ORB was determined as previously reported (Boby et al., 2020). The examined bacteria were grown in LB medium for 24 h before being centrifuged at 5,000 ×g and resuspended in MHB to obtain a concentration of 10^10^ CFU/ml. Further, aliquots of 100 μl of *E. coli* strains (10^10^ CFU/ml) were inoculated onto agar plates with different known doses of ORB. The inoculated plates were incubated at 37°C for 48 h before being visually inspected for growth. The MPC was defined as the lowest antibiotic concentration with no detectable bacterial colonies.

### 2.8 *In vitro* pharmacodynamic model

**FIGURE 1:**
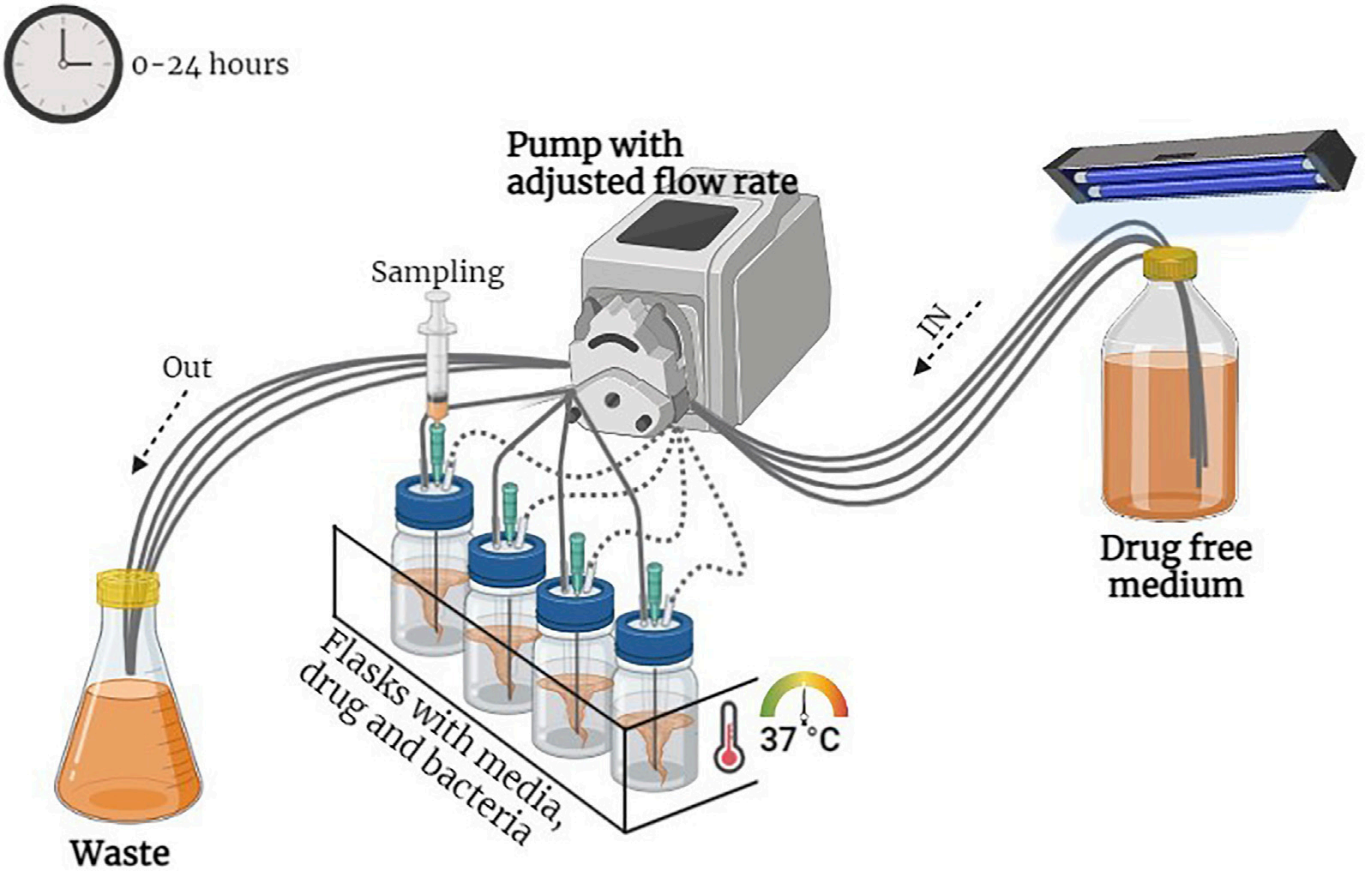
Diagrammatic representation of the pharmacodynamic model.

The model comprised five flasks that were connected with a dilution compartment containing fresh MHB-II broth, a central compartment with either a bacterial culture alone (control growth experiment) or a bacterial culture in a medium containing a drug (killing–regrowth experiment), and an elimination compartment containing waste broth and bacteria. Peristaltic pumps (Masterflex; Cole-Parmer, United States) circulated in one direction, from the dilution compartment to the central compartment and from the central compartment to the elimination compartment, at a flow rate of 5.1 ml/h. The temperature of the system was maintained at 37°C throughout the experiment. An overnight culture of *E. coli* was inoculated in the central unit. Further, ORB, PG, and a combination of both were injected into the central unit which contained 60 ml of MHB-II broth. The PD model is presented in Figure 1.

In the first 24 h of the experiment, multiple samples of bacterium-containing media were collected from the central compartment, and the bacteria were counted throughout the experimental period; samples were collected at 0 h, 1 h, 2 h, 4 h, 8 h, 6 h, 8 h, 12 h, and 24 h. All the samples were diluted in agar saline, poured onto an MH agar plate, and incubated at 37°C for 24 h for the visible bacterial colony count. In this experiment, the MIC of ORB and PG, the sub-MIC of both the drugs, and the individual MIC of the drugs were tested, and treated once at the start. Afterward, the 2^nd^ time treatment was followed by the same protocol with modifications. The first and second groups were treated with the MPC of ORB and the MIC of PG; the third group, a combination of MIC and MPC of both the drugs; the fourth group, sub-MIC and sub-MPC of both the drugs; and, the fifth group was the bacterial control. Samples were collected from each group at 0 h, 1 h, 2 h, 4 h, 8 h, 12 h, 24 h, 26 h, 27 h, 28 h and 48 h. The treatment was administered at 0 h, 12 h, and 24 h, and the total running time was 48 h. Every sample was diluted and poured onto agar plates and incubated at 37°C overnight. The number of colonies was counted, and the CFU was counted.

### 2.9 Quantification of biofilm formation (biofilm-forming assay)

*E. coli* biofilm generation was evaluated as described previously (O'Toole, 2011; Haney et al., 2021), with minor modifications. Briefly, the three-field isolated *E. coli* (KVCC) strains (543,14003061,1423) were inoculated in LB media. *E. coli* ATCC-35218, the wild-type strain of *Pseudomonas aeruginosa* PA-01, and *Staphylococcus aureus* ATCC-29213, which have biofilm-forming properties were the positive controls (Molina et al., 2020; Muhammad et al., 2020). All the bacteria were inoculated in LB media (TSB for *S. aureus*) and incubated at 37°C overnight. The overnight cultures were diluted at 1:100 in two types of freshly prepared media, i.e., LB and LB with 0.5% glucose. In the 96-well flat-bottom microtiter plates, 100 µl of dilutions were dispensed in replicates and incubated at 37°C for 48 h, allowing bacterial attachment and biofilm formation. Following 48 h of incubation, the supernatant was discarded and each well of the 96-well plates was rinsed thrice with sterile distilled water. After rinsing, 125 µl of 0.1% of crystal violet solution (w/v) was added, and the plates were incubated at room temperature for 20 min. After incubation, the plates were washed again with sterile distilled water. Finally, 125 µl/well of 30% acetic acid (v/v) was added to dissolve the dye (Park et al., 2018; Boby et al., 2020). Biofilm formation was measured by determining the OD value.

### 2.10 Minimum biofilm eradication concentration

**FIGURE 2:**
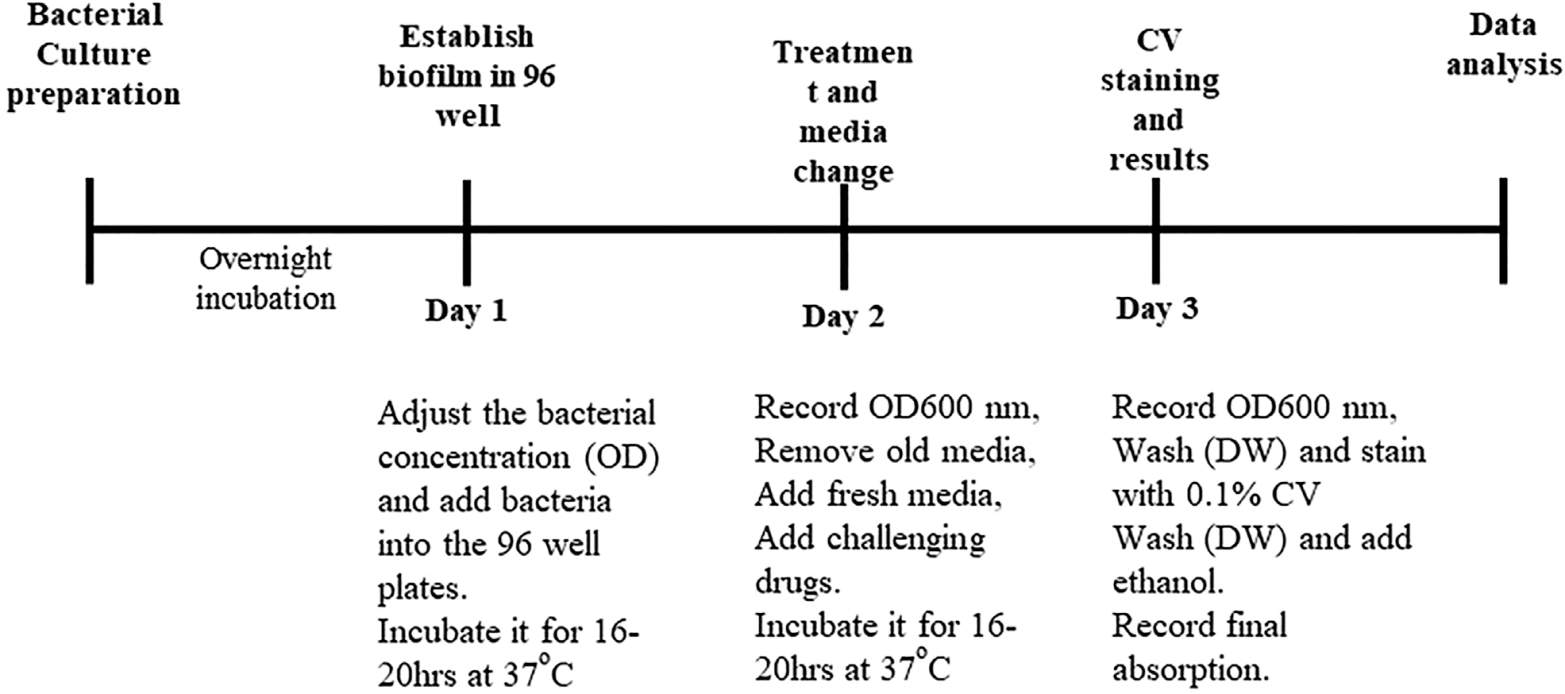
Minimum biofilm eradication concentration protocol diagram.

The minimum biofilm eradication concentration (MBEC) assay was performed as described previously (Haney et al., 2021). In detail, overnight cultures of the six bacteria were prepared in LB media. *E. coli* 543, *E. coli* 1400306, 1,423, and *E. coli* ATCC 35218 were used, and *Pseudomonas aeruginosa* PA-01 and *Staphylococcus aureus* ATCC 29213 were used as positive controls. The experiment protocol is further described in Figure 2.

### 2.11 Swimming and swarming motilities assay

Both the assays were performed according to published protocols with slight modifications (Calvio et al., 2005). For the swarming assay, we added 0.8% LB media, 0.6% agar, and 0.5% glucose mix in DW and autoclaved it. We added the drugs into the autoclaved media (lower temperature), mixed it thoroughly, poured it into the petri dish, and waited for 1 h. Further, 2 µl–5 µl of an overnight grown culture of bacteria was inoculated in the center of the plate. The plates were incubated for 18 h at 37°C. After incubation, the radius of the growth circle was measured. For the swimming assay, 1% LB media with 0.3% agar was added to DW according to the given concentrations by media manufacturers and autoclaved. The drugs were added to the autoclaved media and mixed in thoroughly, and the media was poured into the petri dish and kept aside for 1 h, after which 2 µl–5 µl of the grown culture of bacteria was inoculated at the center of the plate. The plates were incubated for 10 h at 37°C, and the radius of the growth circle was measured (Kim et al., 2003; Zhuang et al., 2016). Both the experiments were divided into four groups: MIC, ½ MIC, a combination of PG and ORB, and control.

### 2.12 Cell viability assay

Cell viability was measured using the 3- (4, 5-dimethyl-2-thiazolyl) -2, 5-diphenyl-2H-tetrazolium bromide (MTT) assay. Confluent cells of a Korean cell line, Raw cell 264.7 (10^5^ cells/ml), were cultured on plates at 37°C and 5% CO_2_ for 24 h. The medium was replaced with a fresh medium containing MIC and sub-MIC of ORB and PG individually and in combination and then incubated overnight at 37°C and 5% CO_2_. Finally, the 0.45% MTT reagent was replaced with a cell culture medium, and the cells were cultured for 4 h, after which 100 μl of DMSO was added. After 5 min, the plate was read at 570 nm. Cell viability was calculated using the following formula:

$$Rate\;of\;viability=\frac{\left(ODcompound-ODblank\right)}{\left(ODcontrol-ODblank\right)}\times 100$$

## 3 Results

### 3.1 Detection by multiplex PCR

**FIGURE 3:**
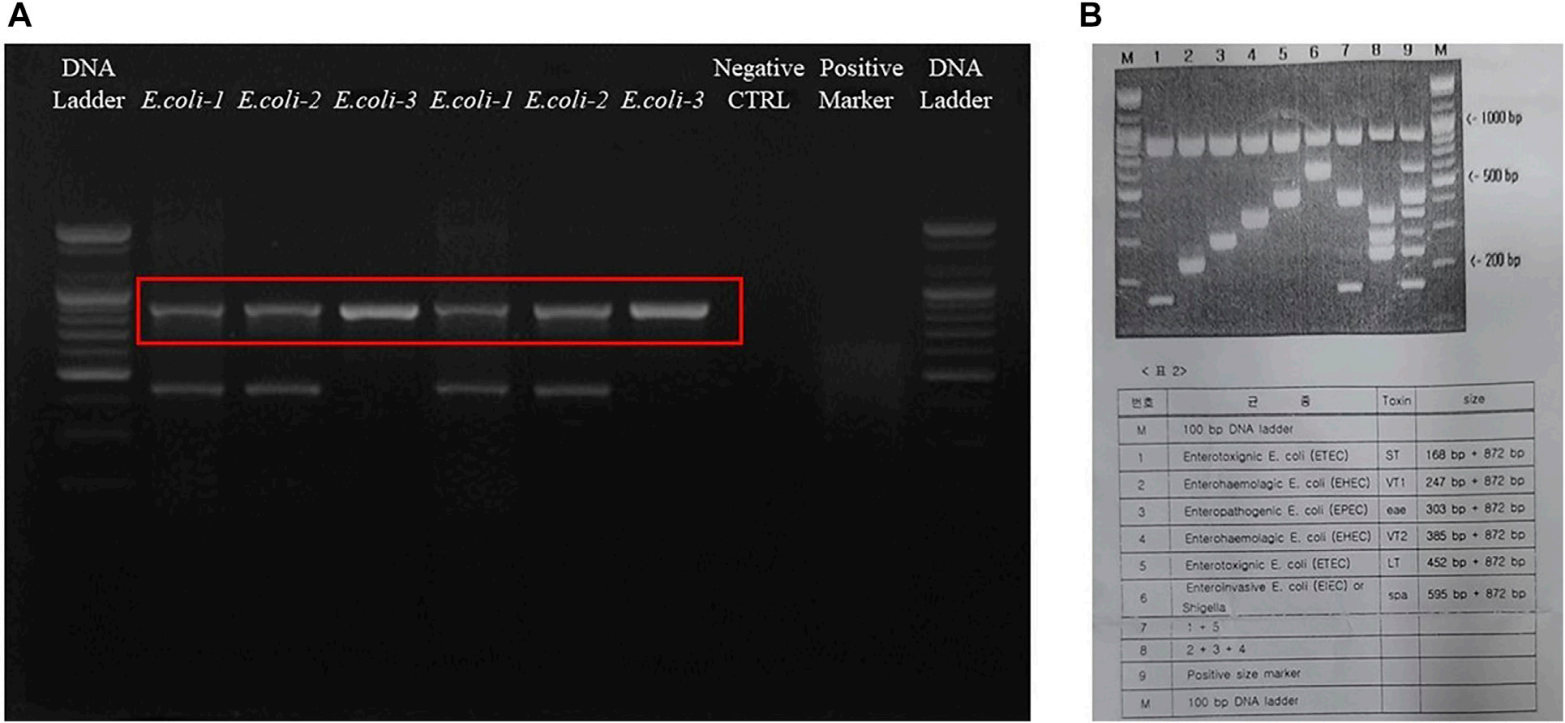
**(A)** The gel electrophoresis image was taken after a 30 min run at 100 v on a 2.5% agarose gel. The first well is the 100 bp DNA ladder, *E. coli*-1 is KVCC-BA 0001423, *E. coli*-2 is KVCC-BK 0000543, and the *E. coli*-3 is KVCC-BA 1400306, which are repeated. The well with no bands is the negative control with only the primer and DW. The 2^nd^ last well contains the positive multiplex marker, and the last well contains the DNA ladder. **(B)** Figure B is the image of the instruction page provided with the kit to identify the exact location with different sizes and types.

The final results were analyzed by comparing the band sizes. The bands are given in Figure 3, which shows that all the bacteria were of the enterotoxigenic *E. coli* (ETEC) (ST) category. Furthermore, specifically, *E. coli*- KVCC-BA 0001423 and *E. coli*- KVCC-BK 0000543 were present in the enterotoxigenic *E. coli* (ETEC) (LT) area, which was inferred by comparing the bands with those in the instructional image (Figure 3B). The results show that *E. coli*- KVCC-BA 0001423 and *E. coli*- KVCC-BK 0000543 both produced both heat-stable (ST) and heat-labile toxins, (LT) but *E. coli*- KVCC-BA 0001423 is the only ST-producing ETEC. The gel electrophoresis image is given in Figure 3A,B is the instruction from the kit.

### 3.2 Antibacterial activity

The final MIC values were interpreted based on the standard guidelines of susceptibility. According to the CLSI standards for ORB, < 4 µg was considered sensitive and > 16 µg (or in some quinolone 32 µg) was considered resistant, and 8 μg, i.e., the midpoint of these two concentrations was considered intermediate. The two *E. coli* strains (BK-543 and BA-1400306) and one quality control strain ATCC 35218 were sensitive to the ORB range of 0.5 μg/ml–2 μg/ml, but one strain was found to have high resistance to ORB at 128 μg/ml. The MIC of PG against all the strains ranged from 312.5 μg/ml to 625 μg/ml. The MBC values were found to be higher than the MIC values in all the strains except for that of the control strain of *E. coli* against ORB, where both MIC and MBC were the same. The results and ratio between MBC and MIC are given in Table 1.

**TABLE 1:** Minimum inhibitory concentration and minimum bactericidal concentration determination of orbifloxacin and propyl gallate.

	Orbifloxacin		Propyl gallate		Orbifloxacin	Propyl gallate
Strains	(MIC) µg/ml	(MBC) µg/ml	(MIC) µg/ml	(MBC) µg/ml	MBC/MIC ratio	MBC/MIC ratio
KVCC-BA 0001423	128 (R)	256	312.5	625	2	2
KVCC-BK 0000543	0.5 (S)	1	312.5	625	2	2
KVCC-BA 1400306	2 (S)	4	625	1,025	2	1.64
ATCC 35218	0.5 (S)	0.5	625	2,500	1	4

*(S-sensitive, R-resistance) *(MIC-minimum inhibitory concentration, MBC- minimum bactericidal concentration).

### 3.3 Static killing activity of drugs

**FIGURE 4:**
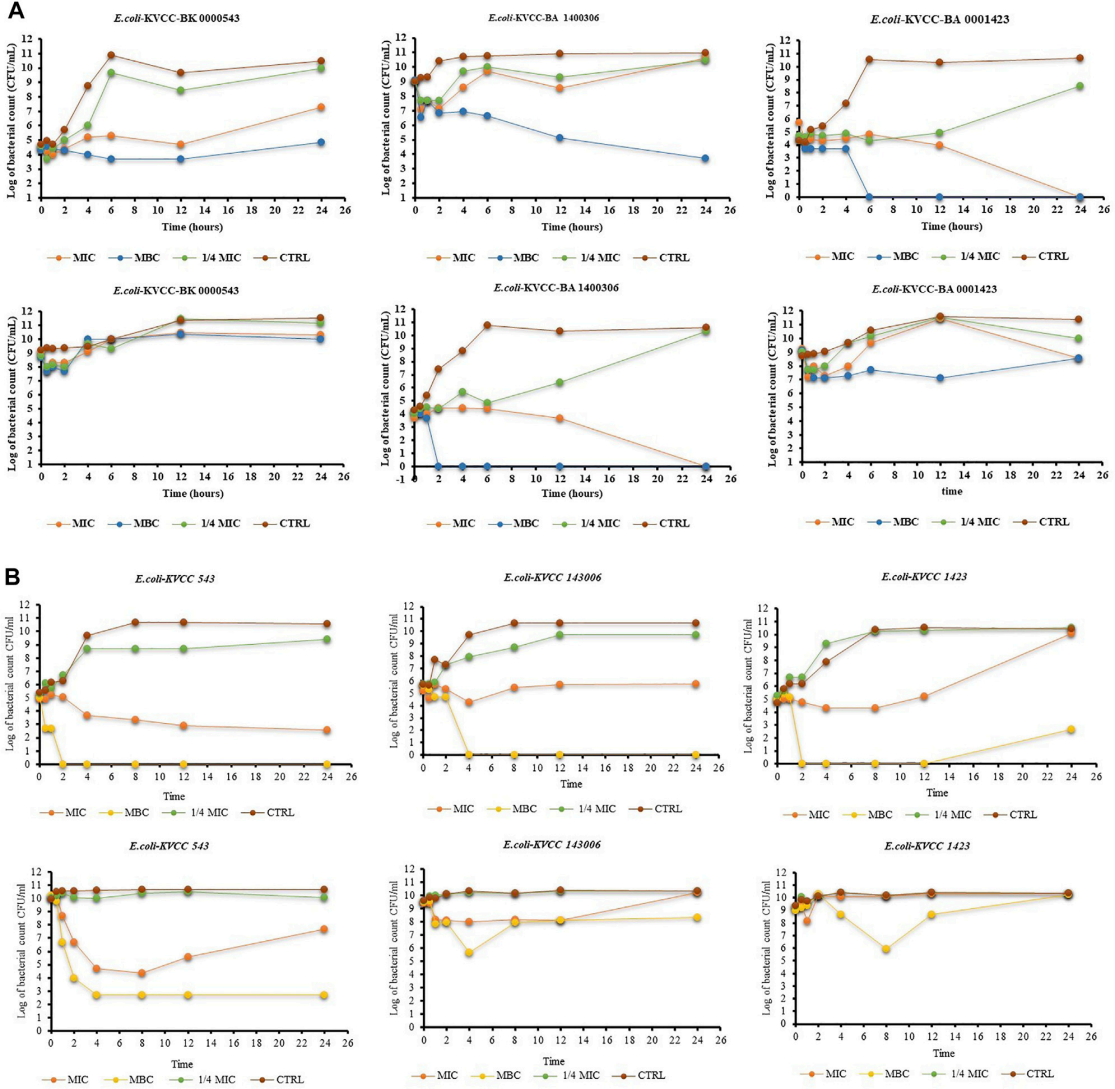
**(A)** Time-kill assay of propyl gallate against *E. coli* (*E. coli*-KVCC-BA 0001423, *E. coli*-KVCC-BK 0000543, *E. coli*-KVCC-BA 1400306) treated with MIC, MBC, and ¼ MIC, and non-treated *E. coli* as a control. **(B)** Time-kill assay of orbifloxacin against *E. coli* (*E. coli*-KVCC-BA 0001423, *E. coli*-KVCC-BK 0000543, *E. coli*-KVCC-BA 1400306) treated with MIC, MBC, and ¼ MIC, and non-treated *E. coli* as a control.

The results of the time-kill activity of PG show its significant antibacterial properties against *E. coli*. The concentrations used were MIC, MBC, and ¼ MIC of PG. One-fourth of MIC showed an antibacterial effect in the first few hours, but the CFU increased with time in all the strains. The MIC of PG against the concentration of 10^5^ CFU showed a bacteriostatic effect until the 12th hour in all the strains. The CFU of *E. coli* 543 started increasing after the 12th hour. In the case of *E. coli* 1400306 and *E. coli* 1,423, the growth showed a continuous declining curve at the MIC. However, the MBC shows a strong killing effect from the start against *E. coli* 1400306 and *E. coli* 1,423, but a static effect was observed in *E. coli* 543. At the higher concentration of CFU 10^9^, there was drug resistance and there was no decrease in the CFU, but the effect was poor. However, the MBC of PG showed a strong static effect even at a higher CFU level of bacteria. The results showed that PG had strong killing activity against this *E. coli* strain at a concentration of 10^5^ CFU, and the bacteria showed resistance at a high concentration of 10^9^ CFU. The results are shown in Figure 4A. The time-kill activity of ORB is shown in Figure 4B. Here, we used MIC, MBC, and ¼ MIC. ¼ MIC showed a poorer antibacterial effect compared to other concentrations, and the graph line showed an increasing trend with time. The MIC value against 10^5^ bacteria showed a bacteriostatic effect until the 12th hour, but the graph line showed an increasing trend at 24 h. The same MIC concentration against 10^9^ bacteria showed a lesser effect in the early h but with time, the effect reduced. The MBC showed a good killing effect against *E. coli* KVCC-1423 at a concentration of 10^5^ CFU. There was no growth after 2 h, but it showed some growth at 24 h at the same concentration; this shows the drug-resistant behavior of this strain.

### 3.4 Synergism between drugs

**FIGURE 5:**
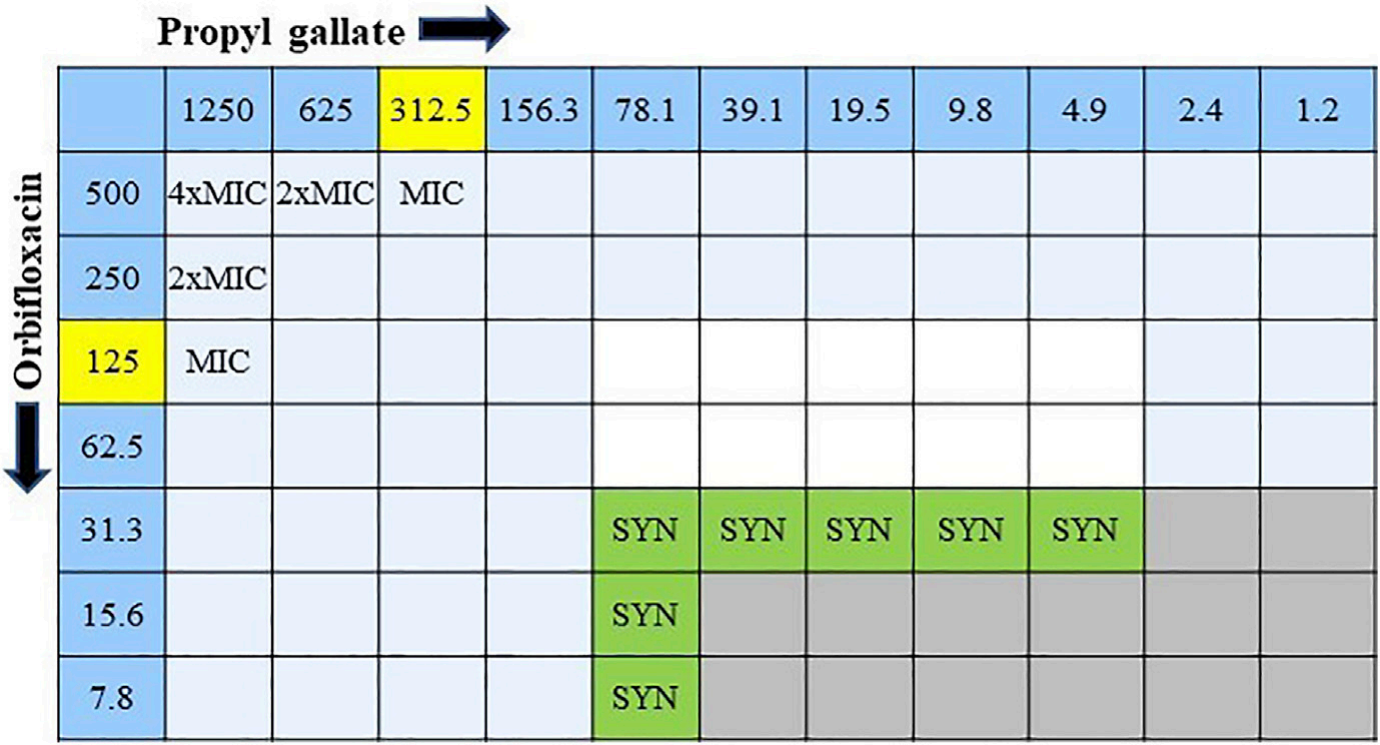
Combination interaction of orbifloxacin with propyl gallate against *E. coli* KVCC 1423 by the checkerboard microdilution method. Gray colored zones indicate bacterial growth, and white colored zones are free of bacteria. (SYN = synergistic effect; MIC = minimum inhibitory concentration).

The combined effect of ORB and PG was determined from the FIC. The combination of ORB and PG showed a strong synergistic effect against *E. coli* 1,423 (Figure 5). The synergistic effect showed that the combination of the drugs reduced the resistance, four times decreased MIC value in ORB and PG from the initial MIC value. The FICI aids in interpreting the effect as synergistic (0 < FICI ≤ 0.5), additive (0.5 < FICI ≤ 1), and indifferent (1 < FICI ≤ 4). The FICI index in our study was lower than 0.5 and showed good synergism between drugs. The data tables are also given in the Supplementary Material.

### 3.5 Mutant prevention concentration

**FIGURE 6:**
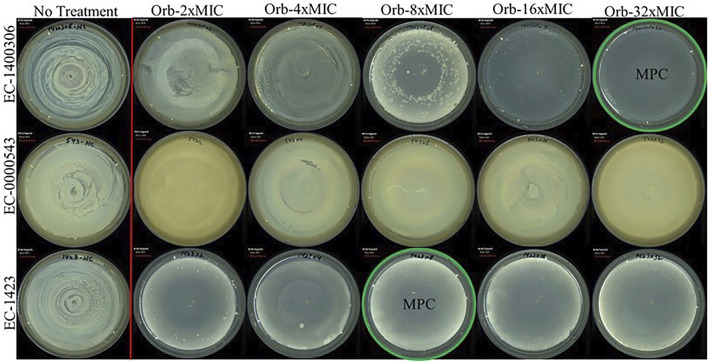
Mutant prevention concentration (MPC) of orbifloxacin against *E. coli* (*E. coli*-KVCC-BA 0001423, *E. coli*-KVCC-BK 0000543, *E. coli*-KVCC-BA 1400306).

The MPC findings of all three bacteria were different (Figure 6). We used a higher concentration that was 32-times the MIC of the drug against the target strains of bacteria. *E. coli* 1,423 showed no growth at 8 × MIC (1,024 µg), which is the MPC concentration of ORB against *E. coli* 1,423, *E. coli* 143,006 showed resistance until 16 × MIC, and there was no growth observed at 32 × MIC, so the MPC of *E. coli* 14700306 32 × of MIC (64 µg). The MIC for *E. coli* 543 was 0.5 µg, and the MPC was 32 × MIC (16 µg), which was the maximum concentration in the experiment, and growth was observed at 32 × MIC.

### 3.6 *In vitro* PD model

**FIGURE 7:**
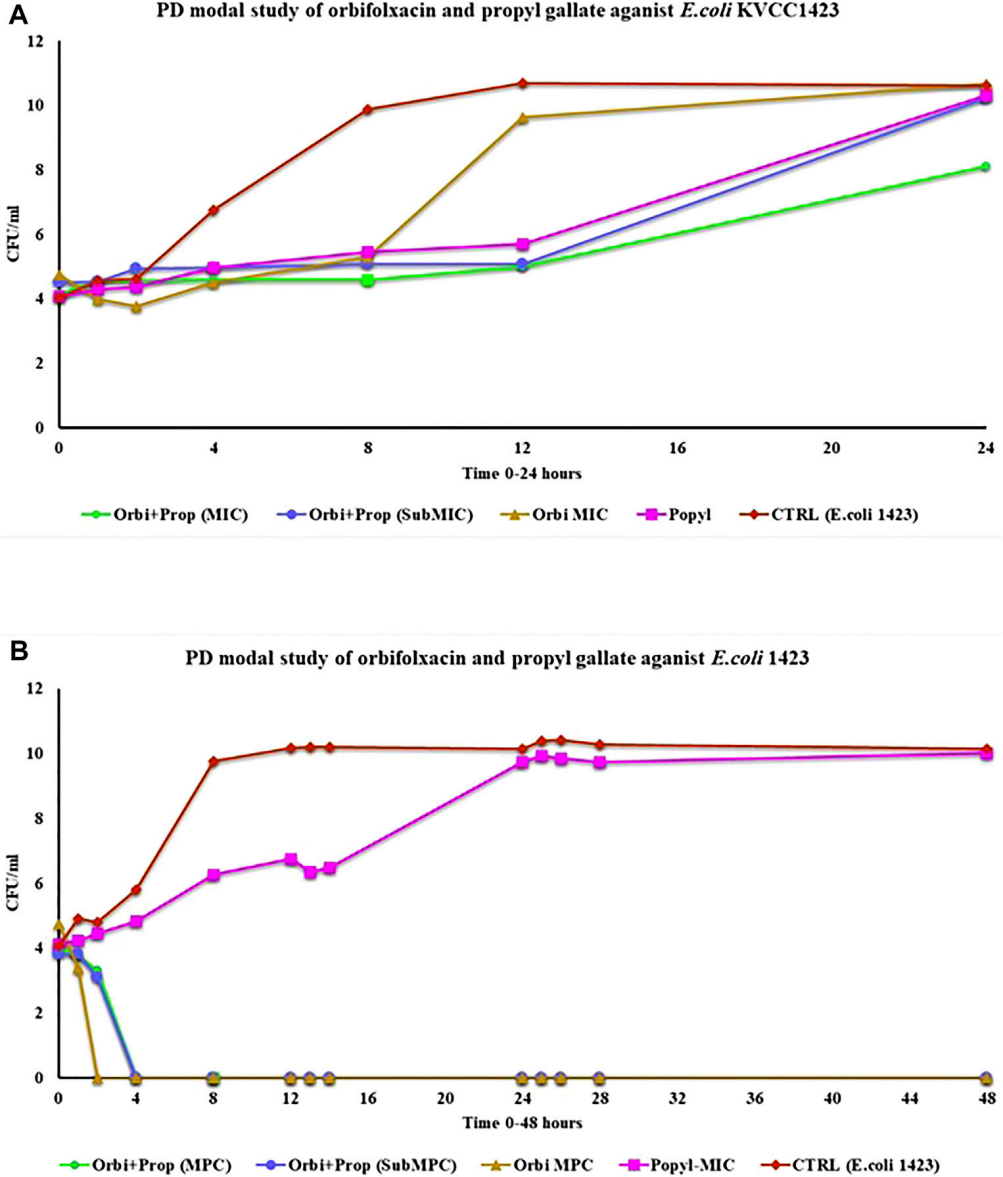
**(A)** Pharmacodynamic model study of MIC of the drugs and the combination of drugs for 24 h against *E. coli* KVCC 1423. **(B)** Pharmacodynamic model study of drugs by using the MPC of orbifloxacin and MIC of propyl gallate (Orbi = orbifloxacin, Prop = propyl gallate).

In the PD model, the static activity of all the groups was observed until 12 h except ORB and the bacterial control. For the ORB MIC (128 μg/ml), resistance was observed until 8 h. After the 8th hour, there was a continuous increase. For the combination of both drugs (MIC, ORB + PG) the significant difference was observed as compered the other groups. In MIC combination the bacterial growth was static until 24 h the effect of the drugs in combination at the MIC was better than that of sub-MIC group and the individual activities of the both drugs. However, throughout the period, the drug medium was replaced by fresh medium at a continues flow rate, and with the removal of the old medium, the drug concentration gradually decreased in the model. The presence of PG with ORB increased the effect of the drugs and reduced the resistance (Figure 7A). The MPC of ORB and the combination of ORB and PG killed the bacteria at 4 h, after which no regrowth was observed until 48 h, but drug treatment was also carried out during this period (Figure 7B). However, in case of PG, the growth was static until 12 h; a decrease was observed in growth until 2 h, after which growth was observed again, and there was no decline observed in the graph line. The control *E. coli* 1,423 showed proper growth.

For further analysis, the sample collected during experiment was cultured into fresh media for overnight. The drug ORB was 2-fold diluted in 96 well plated and bacteria at 10^5^ CFU were added. The 96-well plates were incubated at 37°C. We aimed to check the building of resistance in *E. coli* against ORB after 24 h after exposure to the combination. There was no resistance development in the samples over 24 h.

### 3.7 Quantification of biofilm formation

**FIGURE 8:**
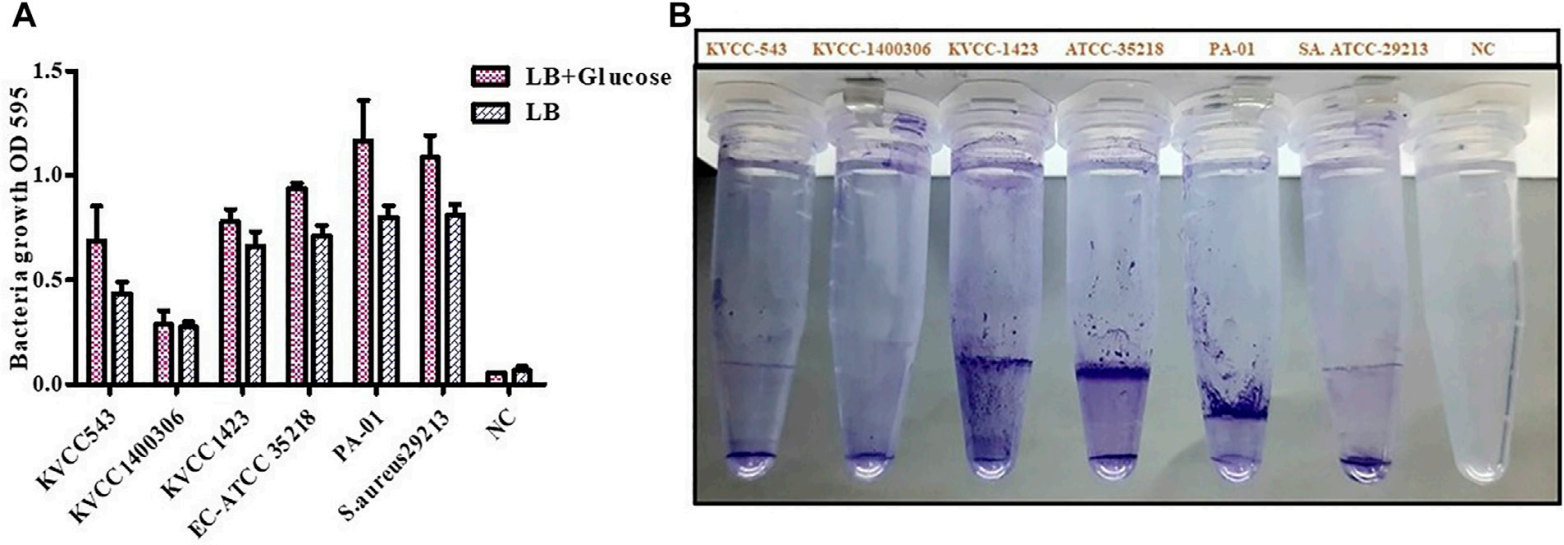
**(A)** Quantification of biofilm formation in *E. coli* (KVCC 543, 1400306, 1,423, ATCC 35218), PA-01, and *S. aureus* ATCC 29213 using an OD value graph. **(B)** Quantification of biofilm formation in *E. coli* (KVCC 543, 1400306, 1,423, ATCC 35218), PA-01 and *S. aureus* ATCC 29213.

Four strains of *E. coli* were inspected for their biofilm formation potential. One strain each of *Pseudomonas aeruginosa* (PA-01) and *S. aureus* (ATCC-29213) known to create biofilms were utilized to examine bacterial growth and biofilm development. To stimulate biofilm development, the LB medium was supplemented with 0.5% glucose. The average bacterial growth in terms of OD_595_ value in LB media was ≥ 0.4; however; bacterial growth in 0.5% glucose-supplemented LB media was from 0.66 to 1.28. If the absorbance was greater than 0.1, the biofilm formation was considered as strong; 0.1–1, moderate; and, ≤ 0.10, absent or weak. Regarding biofilm formation in LB (0.5% glucose) positive controls, the average OD value was 1.16 and 1.08 in the case of PA- 01 and *S. aureus* ATCC 29213 respectively. However, for *E. coli* ATCC 29,213, the average OD value was 0.93, and in our tested strains, the highest OD value was observed for *E. coli* KVCC 1,423, which was 0.77. The OD values of all the strains were slightly lower in the case of LB media. The highest OD values shown by our positive controls PA- 01 and *S. aureus* ATCC 29213 were 0.81 and 0.8, respectively. Therefore, bacterial growth was better in LB with 0.5% glucose, according to the findings of the interaction between biofilm development and planktonic bacteria growth. In our tested strains, *E. coli* KVCC 1423 and KVCC 543 had biofilm-forming properties. The results are shown in Figure 8.

### 3.8 Minimum biofilm eradication concentration activity of drugs

**FIGURE 9:**
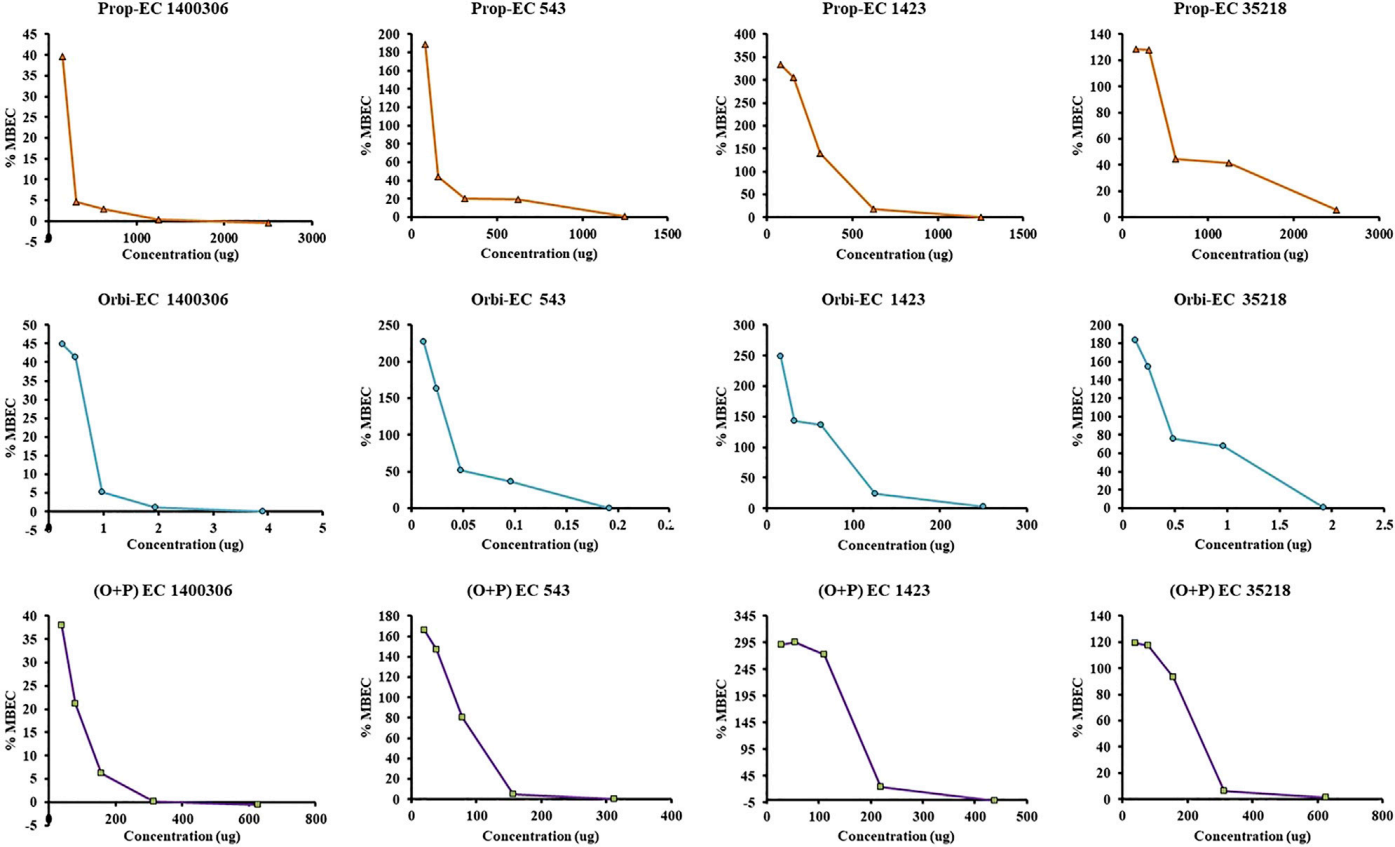
Minimum biofilm eradication concentration (MBEC) activity of individual drugs against four strains of *E. coli*. One axis shows the concentration of the drug and the other represents the percentage MBEC. The ORB + PG (O + P) combination shows better activity as compared with the individual drugs.

In the MBEC experiment, individually ORB, PG, and a combination of ORB and PG were used to target four *E. coli* strains. In the case of individual activity evaluation of ORB and PG, a higher MIC concentration (2 × MIC) was used, but in combination, the MIC of drugs was combined. We found that ORB and PG had good biofilm-eradication properties (Figure 9). For PG, the MBEC was higher than the MIC. However, the MIC of PG against *E. coli* 1400306 and *E. coli* 1,423 showed favorable results, and this is similar in the case of ORB. However, in the case of *E. coli* 543 and EC 35218, good results were observed at 2 × MIC concentration, and this could be because the MIC of the drug was very low against these two bacteria. In combination, the sub-MIC successfully eradicated the biofilm, which shows that the combination of drugs has a synergistic effect against these biofilm-producing bacteria. PA-01 and *S. aureus* ATCC 29213 were used as control strains.

### 3.9 Swim and swarm motilities assay

**FIGURE 10:**
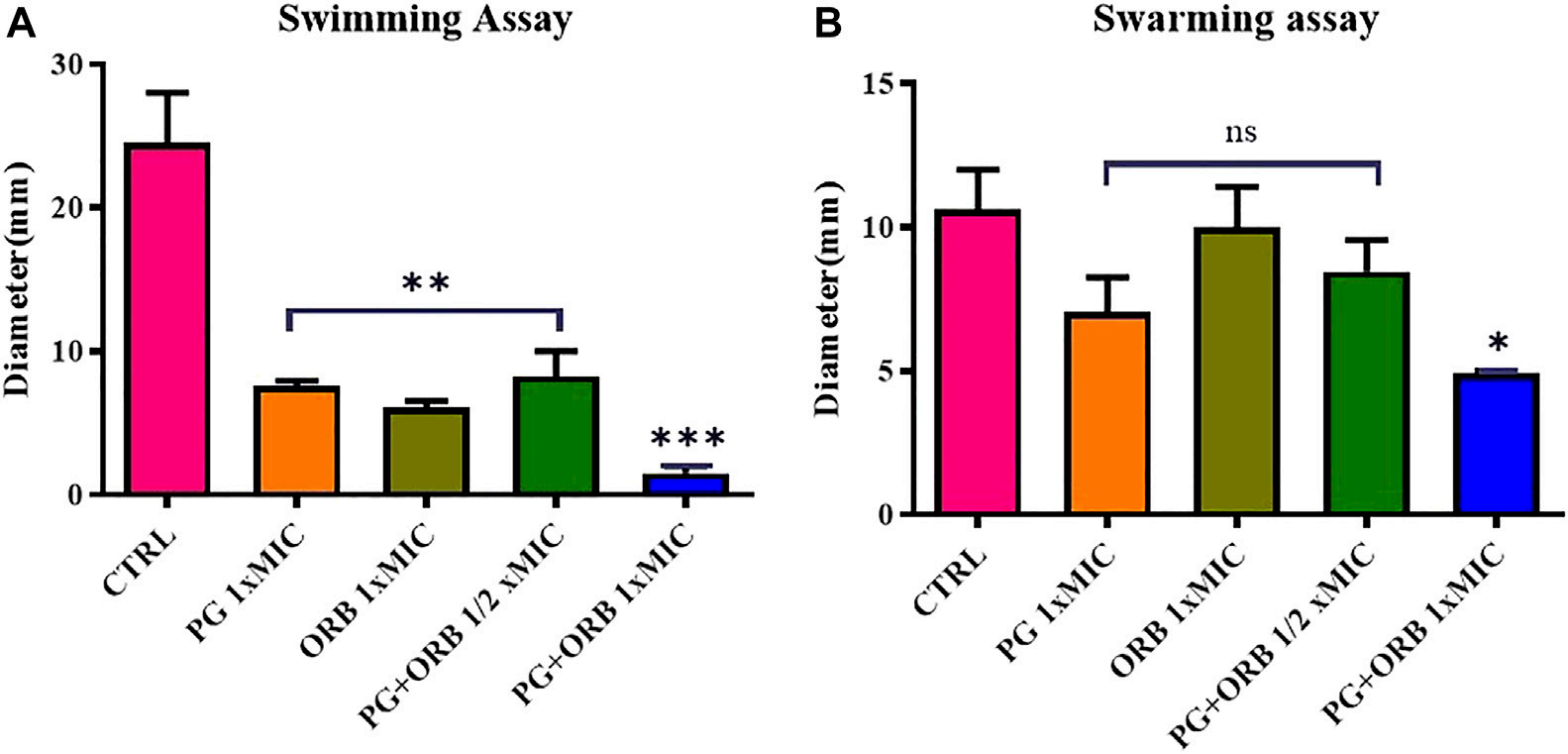
Swimming and swarming motilities assay. Figure **(A)** shows the significant differences of all the groups compared with the control (CTRL). In the swimming assay, a significant difference was observed between individual MIC of the drugs and ½ MIC of the drugs compared with the CTRL, and the combination of both the drugs shows a significant difference compared to all the groups. Figure **(B)** shows the non-significant difference in the three groups; however, the combination shows a significant difference.

The impact of ORB and PG individually and in combination on the swarming and swimming motilities of *E. coli* were assessed. Representative images of swarming and swimming cells (Supplementary Figure), which were either treated or untreated with a combination of antibacterial agents, are shown in Figure 10. The results showed, that there was a significant difference between the groups in the swimming assay. As shown in Figure 10A, the PG and ORB individual activity was almost similar but there was a clear difference between the combination of drugs and the individual activity of drugs. The swarming activity was presented in Figure 10B and showed that the individual activity of both of the drugs is more or less similar. However, a significant difference was observed in the combination group. In swim assay, the combination of ORB and PG restrained the motility of bacteria by 75% and 80% more as compared to the individual activity of ORB and PG, respectively. Similarly, in swarm assay, the combination effects on the bacterial movement were 51% and 30% as compared to the individual activity of ORB and PG, respectively. These findings showed that the swarm and swim motilities of *E. coli* were substantially restrained by the combination. Furthermore, the antibacterial combination at the sub-MIC inhibited the swarming and swimming motilities of *E. coli* more intensely than the antibacterial agents individually could at their 1 × MIC concentrations. The diameters of the swarm and swim zones were measured and are presented in the graph. The images of the plates are provided in the Supplementary Material.

### 3.10 Cell viability assay

**FIGURE 11:**
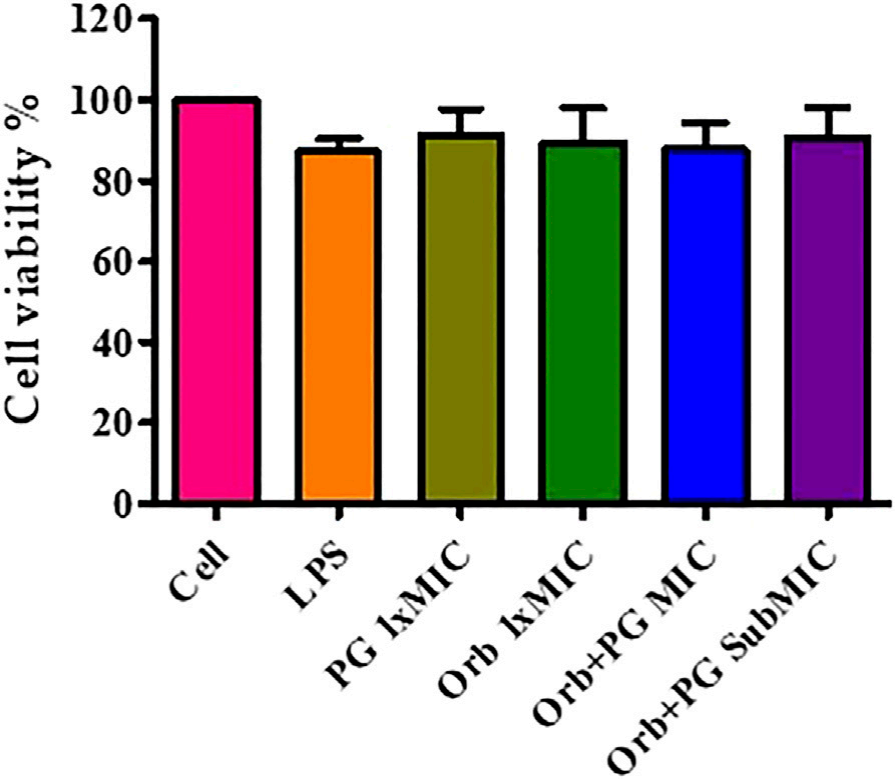
Cell viability assay graph showing more than 80% viability in all the groups.

The cell viability results showed that the viability was more than 80% in all the groups and there was no significant difference observed between the groups (Figure 11). It indicates the absence of negative effects of the drugs and their combination on the cells.

## 4 Discussion

Over the previous few years, *E. coli* are becoming dramatically resistant to fluoroquinolone (FQ), making antibiotic resistance a global threat. However, ETEC resistance is increasing against quinolone and the resistance rate against FQ in swine-isolated bacteria from Europe, Canada, and Japan was 0%–39%, and it was very high in China and Brazil (81% and 54.4%, respectively) (Stapleton et al., 2020; Amsler et al., 2021). Our findings on antibacterial activity show that *E. coli* KVCC 1400306 and KVCC 543 are in the sensitive range, and only KVCC-BA 0001423 was resistant to ORB. Previous studies have reported different MIC values of ORB against a different strain of *E. coli*; the reported MIC range varied from 4 μg/ml to 128 μg/ml (Gebru et al., 2011; Shimizu et al., 2017). Previous literature and the current situation clear the possibilities of high resistance in *E. coli* against antibiotics. A classic *in vitro* method for testing susceptibility is the time-kill kinetics experiment, which measures antibacterial activity during drug exposure with time. PG showed significant killing activity on *E. coli* growth. The complete inhibition and growth inhibition of bacteria indicate that PG has a favorable effect in killing *E. coli*. However, a bacteriostatic effect was observed in the case of *E. coli* 543. The bacterial inhibition and killing potentials of PG are clearer after this. In the case of ORB, the killing activity against all the strains was definite. However, in the case of resistant strains, we observed growth after 12 h, which reflects the drug-resistant behavior of this strain.

The checkerboard assay revealed that the combination of PG and ORB has a strong synergistic effect, ranging from several dilutions below the MIC. The FICI index was lower than 0.5, which is an indication of strong synergism. Especially in the case of resistant strains, approximately 74% of the MIC decreased in both the drugs. Furthermore, this combination will help to decrease the resistance in bacteria that are highly resistant to ORB. The MPC was determined with log 10^10^ CFU/ml bacteria which were spread on Mueller–Hinton agar supplemented with different ORB concentrations (from 1 × to 32 × MIC value). In this assay, the MPC recorded against *E. coli* 143,006 was 64 μg, and MPC was higher against *E. coli* KVCC 1,423, which was 1,024 µg. *E. coli* 543 showed clear growth at 32 × MIC; therefore, we considered an MPC greater than 32 × in this case. The ratio between MIC and MPC against *E. coli* 1400306 and 1,423 were 1:32 and 1:8, respectively.

The synergistic effect of the combination was evaluated with the pharmacodynamic model (PD model). The drug-kill activity in dynamic activity is more challenging as compared to the static-killing activity. Here, the drug media is continuously removed from the model and replaced with fresh media. However, bacterial growth increases continuously. In this challenging assay, the MIC of the combination showed a response against the resistant strain, and a static effect was observed until 24 h, which indicates the long-term effect of this combination. To observe more efficient killing effects of this combination, the MPC of ORB was combined with the MIC of PG. The results showed the killing of all the populations of bacteria only in the early hour at sub-MIC concentrations. Meanwhile, the sample collected during the experiment was actually the bacterium that was exposed to the drug, tested again through the microdilution method with the MIC concentration to check the resistance development after drug exposure; however, we did not detect resistance development after exposure to the combination.

The biofilm quantification assay was performed to assess the biofilm-forming properties of our target strains as these properties are involved in resistance. In this assay, we used one strain each of *Pseudomonas aeruginosa* and *S. aureus*, which have strong biofilm-forming properties, to compare our results. In our results findings, *E. coli* KVCC 1,423 and 543 showed strong biofilm-forming properties. However, in the MBEC assay, we found that PG, as well as ORB, eradicated biofilm formation at a high concentration. However, sub-MIC of the combination of two drugs successfully eradicated biofilm production in bacteria.

Motility is a pathogenic feature of bacteria that is associated with the dispersion and mobility of bacterial cells. Bacterial cells use this harmful characteristic to evade the host immune response. Flagella are known to drive swimming motility. The flagella are also involved in the swarming movement and biofilm production (Taguri et al., 2004; Zhuang et al., 2016). According to previous research, swarming cells are more resistant to certain antibiotics than biofilm-forming cells (Kim et al., 2003; Lai et al., 2009). The treatment with the combination significantly inhibited swarming and swimming motilities in *E. coli*, implying that this antibacterial combination may affect flagella-associated processes, specifically flagella biosynthesis, chemotaxis, and rotation, which could lead to a decrease in swarming and swimming motilities.

A cell viability assay was performed to identify the effect of the combination on cells. However, we did not find any negative effects of the combination and the individual drugs, and the percentage of living cells was more than 80% in all the groups.

*E. coli* develops several mutational changes and improvements that affect the performance of fluoroquinolones in reaching the precise target location. Resistance is most commonly developed in target enzymes, specifically, DNA topoisomerase II (DNA gyrase) and topoisomerase IV, which are known as the principal processes by which resistance arises (Karczmarczyk et al., 2011). The special qualities of fluoroquinolones are that they occupy the ligation of DNA during the process of replication, and DNA degeneration kills the cells. The other significant aspect is permeability, which limits medication absorption and functions as a primary barrier. The factor also contributes to the drug extrusion of fluoroquinolones resistance via efflux pumps, some of which may have a broad substrate specificity; or one of the more recently reported plasmid-mediated quinolone resistance (PMQR) pathways. (Hopkins et al., 2005; Strahilevitz et al., 2009; Karczmarczyk et al., 2011). However, given current studies and the synergistic impact of our findings, we can hypothesize the mechanism of this combo. The bacterial cell envelope is a complicated multi-layered structure that protects these organisms from their uncertain and frequently hostile environment. However, this is also bacteria’s first line of defense against antimicrobials. The majority of bacteria acquire modifications in their outer membrane, making it impossible for the antibiotic to enter and bind to the exact location.

**FIGURE 12:**
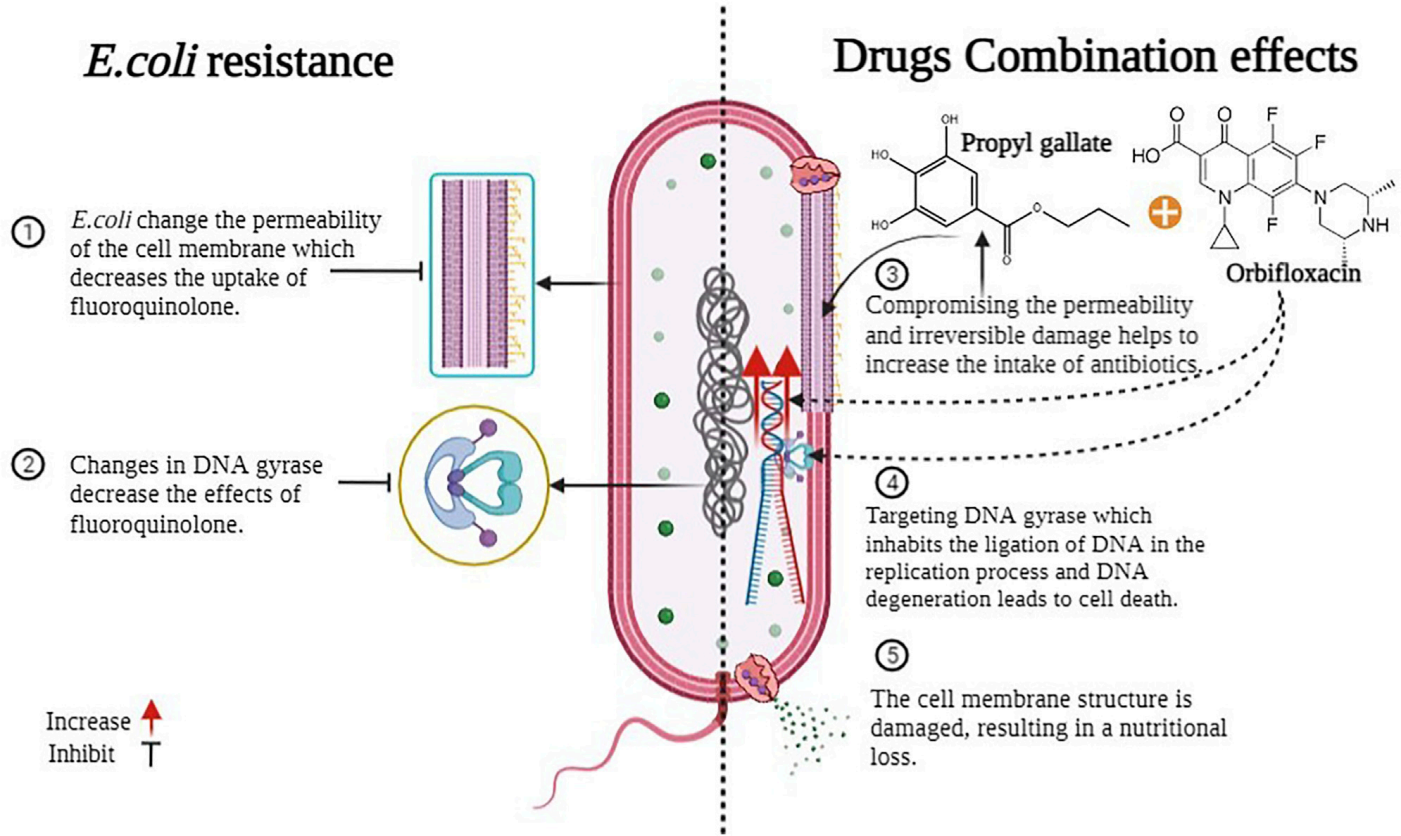
Mechanism of antimicrobial activity combination.

Gallates impair permeability and cause bacterial membrane serious harm (*E. coli*) (Naqvi et al., 2019). The outer layers’ responsibility is for the shape and fluid transport within and without bacteria (Silhavy et al., 2010). Porins (hydrophilic channels) are found in the outer membrane of Gram-negative bacteria and prevent things from entering the cell. However, certain drugs (such as PG) dissolve the lipopolysaccharide layer, reducing membrane permeability and causing nutrients to spill, and harming bacterial growth (Borges et al., 2013; Naqvi et al., 2019). As a result of the damage, the repair mechanism and various other processes in bacteria are activated. Because of their propensity to create ionic and protonic connections and deactivate various biological proteins, such as receptors, ion channels, enzymes, and carriers, hydroxyl groups have an impact on all of these activities (Wang, 2007). Meanwhile, antibiotic absorption may increase due to membrane permeability degradation, and an increase in the number of medications inside the bacterium would enhance the likelihood of the drug attaching to its target location. Figure 12 helps us to understand all this. However, this is hypothetical, and we will be able to grasp the process using references to past research and present results. However, understanding the mechanism of bacterial drug resistance and the activity of antimicrobials is still quite challenging, and many realities need to be revealed.

Based on the results of this investigation, we can conclude that the combination of PG and ORB is effective, promising, and novel for eradicating pathogenic *E. coli*. The combination of PG and ORB effectively suppressed *E. coli* resistance more than either PG or ORB alone, which is critical for improving antibiotic efficacies and developing novel antibacterial combinations to reduce the pathogenic effects of *E. coli* as well as its impact on livestock and public health.

## 5 Conclusion

In conclusion, we demonstrated the antibacterial activity of PG and the combination of PG with ORB against *E. coli in vitro*. PG could inhibit bacteria growth in both cases, both individually and in combination with ORB. Furthermore, the efficacy of the drugs enhances and MICs reduce when combined; thus, showing a synergistic effect. The combination inhibited *E. coli* biofilm development. The combination of PG and ORB showed the ability to confront bacterial mobility without having any harmful effects on the cell. This shows that PG and ORB can diminish antimicrobial resistance while simultaneously combating the component that contributes to resistance. However, further *in vivo* pharmacokinetics and pharmacodynamics are warranted before conducting a preclinical study.

## Data Availability

The study’s original contributions are available in the article/Supplemental Material; additional inquiries can be directed to the corresponding authors.
