# Supplementary material for: A pharmacodynamic investigation to assess the synergism of orbifloxacin and propyl gallate against Escherichia coli
Source: Front Pharmacol. 2022 Sep 15;13:989395. doi: 10.3389/fphar.2022.989395 (PMC9521280; doi:10.3389/fphar.2022.989395)
Supplement: Supplementary file 1 [file DataSheet1.docx]

Supplementary Material

# Supplementary Data Tables

**Table 1: Table 1.A, 1.B and 1.C represent the FIC of *E.coli* KVCC 1423, *E.coli* KVCC 1400306, *E.coli* 543.**

**Table 1.A**

| **Orbifloxacin** | **Propyl gallate** | **FICI** | **S,I,A** |
| --- | --- | --- | --- |
| 0.03125 | 0.00488281 | 0.265625 | synergistic |
| 0.03125 | 0.00976563 | 0.28125 | synergistic |
| 0.03125 | 0.01953125 | 0.3125 | synergistic |
| 0.03125 | 0.0390625 | 0.375 | synergistic |
| 0.03125 | 0.078125 | 0.5 | synergistic |
| 0.015625 | 0.078125 | 0.375 | synergistic |
| 0.0078125 | 0.078125 | 0.3125 | synergistic |

**Table 1.B**

| **Propyl gallate** | **FIC-PG** | **Orbifloxacin** | **FIC-Orb** | **FICI** | **S,I,A** |
| --- | --- | --- | --- | --- | --- |
| 0.015625 | 0.25 | 0.00195313 | 0.00390625 | 0.253906 | synergistic |
| 0.015625 | 0.25 | 0.00390625 | 0.0078125 | 0.257813 | synergistic |
| 0.015625 | 0.25 | 0.0078125 | 0.015625 | 0.265625 | synergistic |
| 0.015625 | 0.25 | 0.03125 | 0.0625 | 0.3125 | synergistic |
| 0.015625 | 0.25 | 0.03125 | 0.0625 | 0.3125 | synergistic |
| 0.0078125 | 0.125 | 0.03125 | 0.0625 | 0.1875 | synergistic |
| 0.00390625 | 0.0625 | 0.0625 | 0.125 | 0.1875 | synergistic |
| 0.00390625 | 0.0625 | 0.125 | 0.25 | 0.3125 | synergistic |

**Table 1.C**

| **Propyl gallate** | **FIC-PG** | **Orbifloxacin** | **FIC-Orb** | **FICI** | **S,I,A** |
| --- | --- | --- | --- | --- | --- |
| 0.015625 | 0.5 | 0.25 | 0.125 | 0.625 | additive |
| 0.0078125 | 0.25 | 0.25 | 0.125 | 0.375 | synergistic |
| 0.0078125 | 0.25 | 0.5 | 0.25 | 0.5 | synergistic |
| 0.0078125 | 0.25 | 1 | 0.5 | 0.75 | additive |

# Supplementary Figures


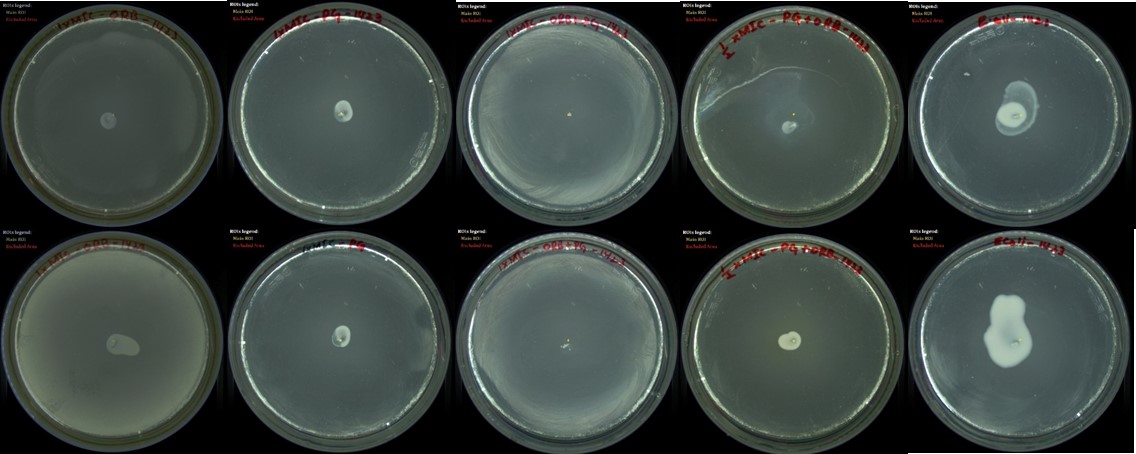


**Supplementary Figure 1.** Swarming assay agar plates shows the growth and motility of bacteria. However, the motility is inhibited by the combination can be orbifloxacin and propyl gallate combination.


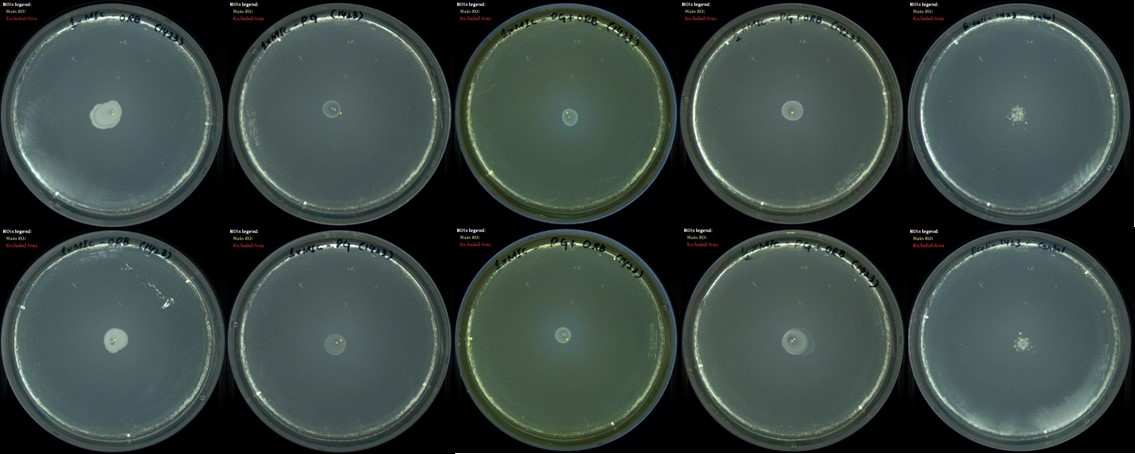


**Supplementary Figure 2.** Swimming Assay agar plates show the growth and motility of bacteria. The motility encountered by the combination can be orbifloxacin and propyl gallate combination.
